# Integrated Transcriptomic and Proteomic Analysis in the Roadmap of the Xylem Development Stage in *Populus tomentosa*

**DOI:** 10.3389/fpls.2021.724559

**Published:** 2021-11-04

**Authors:** Chong Zhang, Jiaxue Zhang, Yadi Liu, Xiatong Liu, Xiaorui Guo, Hui Li, Di Liu, Hai Lu

**Affiliations:** ^1^Beijing Advanced Innovation Center for Tree Breeding by Molecular Design, Beijing Forestry University, Beijing, China; ^2^College of Biological Sciences and Biotechnology, Beijing Forestry University, Beijing, China

**Keywords:** xylem development, transcriptome, proteome, transcriptional regulation, *Populus tomentosa*

## Abstract

Xylem development plays an important role in the wood formation of plants. In this study, we found that xylem development was a rapid thickening process characterized by initially rapid increases in the number of tracheary elements and fiber cells and the thickness of the secondary walls that later plateaued. Transcriptome analysis showed that the xylan and lignin biosynthetic pathways, which are involved in the early rapid thickening of the xylem, were mainly upregulated in the second month. The expression of a total of 124 transcription factors (TFs), including 28 NAC TFs and 31 MYB TFs, peaked in 2- and 3-month-old plants compared with 1-month-old plants. Based on previous studies and the key *cis*-acting elements secondary wall NAC-binding elements, secondary wall MYB-responsive elements, W-box and TGTG[T/G/C], 10 TFs related to xylem development, 50 TFs with unknown function, 98 cell wall biosynthetic genes, and 47 programmed cell death (PCD) genes were used to construct a four-layer transcriptional regulatory network (TRN) with poplar NAC domain TFs to characterize the transcriptional regulation of cell wall biosynthesis and PCD in *Populus tomentosa*. The proteome revealed that post-transcriptional modification may be widely involved in lignification development. Overall, our results revealed that xylem development is a rapid thickening process in *P. tomentosa*, and expression patterns varied temporally from cell division to cell death.

## Introduction

Wood in perennial angiosperms is formed by the differentiation of angiogenic layers into xylem cells. In angiosperms, it is mainly composed of axially elongated vascular elements and fibers and radially elongated ray cells (Mellerowicz et al., 2001). The formation of wood involves many developmental processes, starting from the differentiation event in which the vascular cambium develops into xylem mother cells, and then xylem mother cell differentiation, cell differentiation, cell expansion, secondary wall thickening, and other processes to form heartwood (Schrader et al., 2004). In the past few decades, molecular and genomic studies have revealed various wood-associated biosynthetic genes that play a role in the biosynthesis of cellulose, xylan, glucomannan, and lignin (Mellerowicz and Sundberg, 2008).

The secondary cell wall is a cell structure produced after the specific differentiation of plant cells, and the thickening of the secondary cell wall is regulated by various factors. Secondary wall formation-related genes mainly comprise a series of NAM, ATAF1/2, and CUC2 (NAC) and v-myb avian myeloblastosis viral oncogene homolog (MYB) transcription factors (TFs) forming a hierarchical network that gradually regulates the synthesis of cellulose, hemicellulose, and lignin during secondary wall formation (Nakano et al., 2015). Previous studies indicate that the biosynthesis of the xylem cell wall of *Arabidopsis* is mediated by a three-layer transcriptional regulation model (Ko et al., 2014; Xiao et al., 2021). Recent studies have found a similar regulatory network in bananas, in which NAC and MYB TFs forming a complex transcriptional network that regulates secondary wall deposition (Negi et al., 2017, 2018). Compared with *A. thaliana*, the thickening of the secondary wall has a more complex four-level hierarchical transcriptional regulatory network (TRN) in *Populus trichocarpa*. Previous research has shown that *PtrWND2B* and *PtrWND6B*, which are functional orthologs of *Arabidopsis SND1* and *VND7*, function as master switches activating the secondary wall biosynthetic program in fibers and vessels (Zhong et al., 2010, 2011). In poplar, *PtrMYB2*, *PtrMYB3*, *PtrMYB20*, and *PtrMYB21* are also functional orthologs to *AtMYB46* and *AtMYB83*, and direct targets of the poplar secondary wall *NAC* master regulators *PtrWNDs* (Zhong et al., 2013). In addition, *PtrMYB74* is directly targeted by *PtrMYB21*, and functionally homologous to *AtMYB50*, which targets downstream TFs including *PtrMYB59*, *PtrMYB88*, *PtrMYB90*, *PtrMYB93*, *PtrMYB161*, *PtrMYB174*, *PtrNAC105*, *PtrNAC123*, *PtrNAC125*, and *PtrWBLH1/2*, involved in the regulation of secondary wall thickening (Chen et al., 2019). These studies indicated that the TRN underlying the thickening of the secondary walls of the xylem is highly conserved; however, the large size of the poplar genome has made it difficult to study the TRN. Comparative studies of poplar with *Arabidopsis* are needed.

Several transcriptomic studies of wood-forming tissue in *Populus* have shown that gene expression is tightly controlled (Sundell et al., 2017; Ning et al., 2018; Abreu et al., 2020). However, few studies have characterized the expression patterns of genes and proteins involved in the different stages of xylem development. Here, we analyzed the developmental phenotype, transcriptome, and proteome of the xylem in 1, 2, and 3-month-old plants, referred to as WT-1M, WT-2M, and WT-3M plants, respectively. Our results showed that xylem development is a rapid thickening process in *Populus tomentosa*, and distinct expression patterns were observed from cell division to cell death.

## Results

### Development of Stem Xylem

**FIGURE 1:**
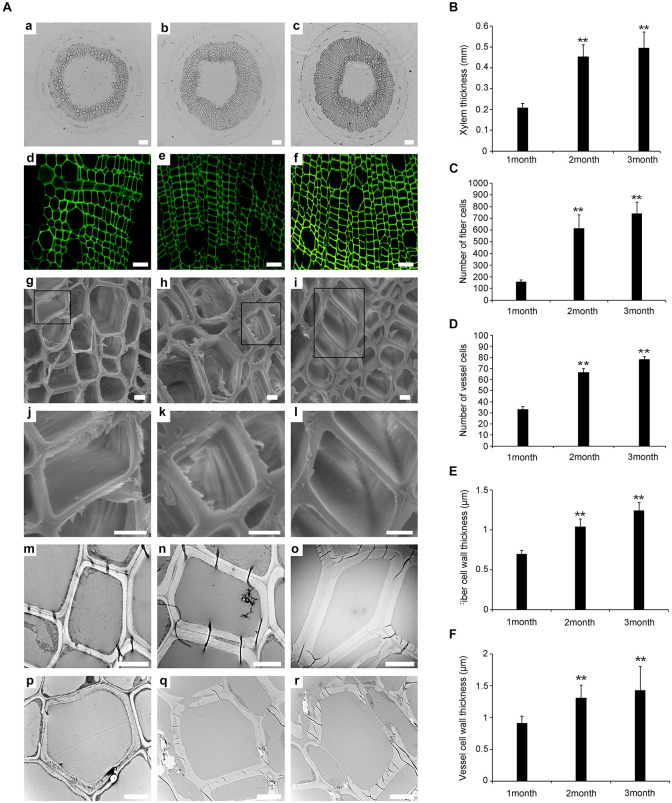
Change in stem xylem development in *P. tomentosa*. **(A)** Paraffin section (**a–c**, scale bar = 200 μm), confocal laser scanning microscope images (**d–f**, scale bar = 50 μm), scanning electron micrographs (**g–l**, scale bar = 5 μm), and transmission electron micrographs (**m–r**, scale bar = 5 μm) of stem transverse sections at the three developmental stages. **(a,d,g,j,m,p)** The fourth internode of 1-month-old plants. **(b,e,h,k,n,q)** The fourth internode of stage of 2-month-old plants. **(c,f,i,l,o,r)** The fourth internode of 3-month-old plants. **(j,k,l)** Are enlarged view of **(g,h,i)**, respectively. **(B)** Xylem thickness of the fourth internode from the base upward in the three developmental stages. **(C)** The number of fiber cells of the fourth internode. **(D)** The number of vessel cells of the fourth internode. **(E)** Fiber cell wall thickness of the fourth internode. **(F)** Vessel cell wall thickness of the fourth internode. The data are means ± SD (*n* = 60). **P < 0.01.

Previous studies have shown that the development of the xylem plays an important role in wood formation in plants (Fukuda, 2016). We determined the number of xylem cells and the thickness of xylem cell walls at different plant ages (1, 2, and 3 months). Paraffin sectioning and confocal laser scanning microscopy revealed that there were significant differences in the development of the xylem during the three time points (Figures 1Aa–f). The thickness of the xylem was greater in WT-2M than in WT-1M (Figure 1B). The number of tracheary elements (TEs) and fiber cells was significantly increased in WT-2M plants than in WT-1M (Figures 1C,D). The difference in the number of TEs and fiber cells between WT- 3M and WT-2M plants was small. These results showed that WT-2M plants were in a period of rapid xylem formation.

Scanning electron microscopy (SEM) and transmission electron microscopy (TEM) analysis were used to study the secondary wall thickening of fiber cells over the 3 months (Figures 1Ag–r). We calculated the thickness of the secondary wall using Image software, and the results showed that the thickness of the secondary wall of the xylem fiber cells was.57 ± 0.02 μm, 1.05 ± 0.06 μm, and 1.24 ± 0.1 μm in WT-1M, WT-2M, and WT-3M plants, respectively. While the thickness of the secondary wall of TE cells was 0.91 ± 0.11 μm, 1.31 ± 0.2 μm, and 1.43 ± 0.37 μm in WT-1M, WT-2M, and WT-3M plants, respectively (Figures 1E,F).

To investigate xylem cell PCD during xylem development, we examined cellular degradation using TEM. No degradation of cellular components, including mitochondria, plastids, vesicles, and membrane-like vesicles, could be observed in mature fiber cells, and few cellular components were observed in the TE cells of WT-1M. Only a few cellular components, such as mitochondria, plastids, and non-degraded nuclei were observed in the cytoplasm of fiber cells in WT-2M plants. The cellular components had completely disappeared in the fiber cells of WT-3M plants. In TE cells of WT-2M or WT-3M plants, the cellular contents had completely disappeared. These results showed that WT-2M plants were in a period of rapid xylem growth.

### Transcriptome Analysis of Differentially Expressed Genes During the Xylem Development

**FIGURE 2:**
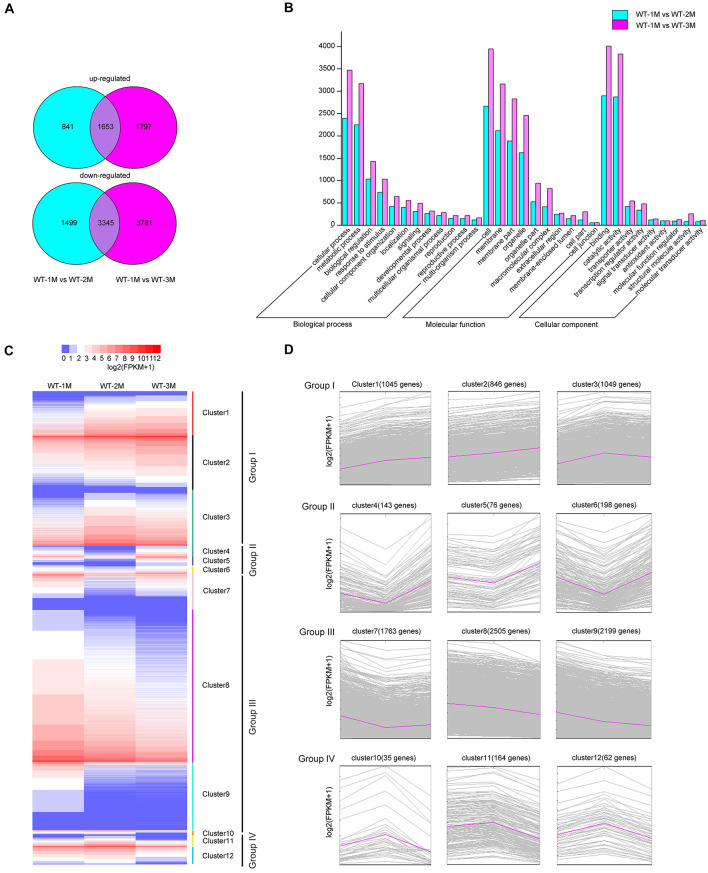
Analysis of differentially expressed genes in WT-2M and WT-3M plants compared with WT-1M plants. **(A)** Numbers of upregulated and downregulated DEGs in WT-1M vs. WT-2M and WT-1M vs. WT-3M. *P* < 0.01, fold change ≥ 1 or ≤ –1. **(B)** Numbers of DEGs of “Biological process,” “Cellular component,” and “Molecular function” in different functional categories in WT-1M vs. WT-2M and WT-1M vs. WT-3M. **(C)** Hierarchical cluster analysis of DEGs of WT plants at three different months. The color scale bar on the top indicates the expression level. The values given in the legend are log2(FPKM + 1). **(D)** 12 clusters (clusters 1–12) were obtained by the soft clustering method for DEGs. The horizontal axis represents the three months (months 1, 2, and 3). The vertical axis represents fold changes in gene expression.

To characterize xylem development at the molecular level, we performed transcriptomic analysis using total RNA from the xylem of the fourth stem section from WT-1M, WT-2M, and WT-3M plants. We identified 12,916 genes with significant differential expression (fold change ≥ 1 or ≤ –1, and corrected *P*-value < 0.01) in at least one of the three time points. Compared with WT-1M plants, 2,494 and 4,844 genes were upregulated and downregulated in the WT-2M plants, respectively (Figure 2A). Compared with WT-1M plants, 3,450 genes and 7,126 genes were upregulated and downregulated in WT-3M plants, respectively. The metabolic process and cellular process in “Biological process,” membrane and organelle in “Cellular component,” and binding and catalytic activity in “Molecular function” were enriched gene ontology (GO) terms in WT-2M and WT-3M plants compared with WT-1M plants (Figure 2B).

Hierarchical cluster analysis of the differentially expressed genes (DEGs) was performed, and DEGs were divided into 12 clusters based on their expression patterns. We divided the 12 clusters into four groups with similar expression patterns (Figure 2C). In group I, upregulated genes were clustered together with 2,940 genes (clusters 1, 2, and 3 included 1,045, 846, and 1,049 genes, respectively). Group III included 6467 downregulated genes, while clusters 7, 8, and 9 included 1,763, 2,505, and 2,199 genes, respectively (Figure 2D). In group II, 417 genes were downregulated and then upregulated, whereas group IV included 251 genes that were upregulated and then downregulated (Figure 2D). These results indicated that DEGs were mainly downregulated during xylem development.

**FIGURE 3:**
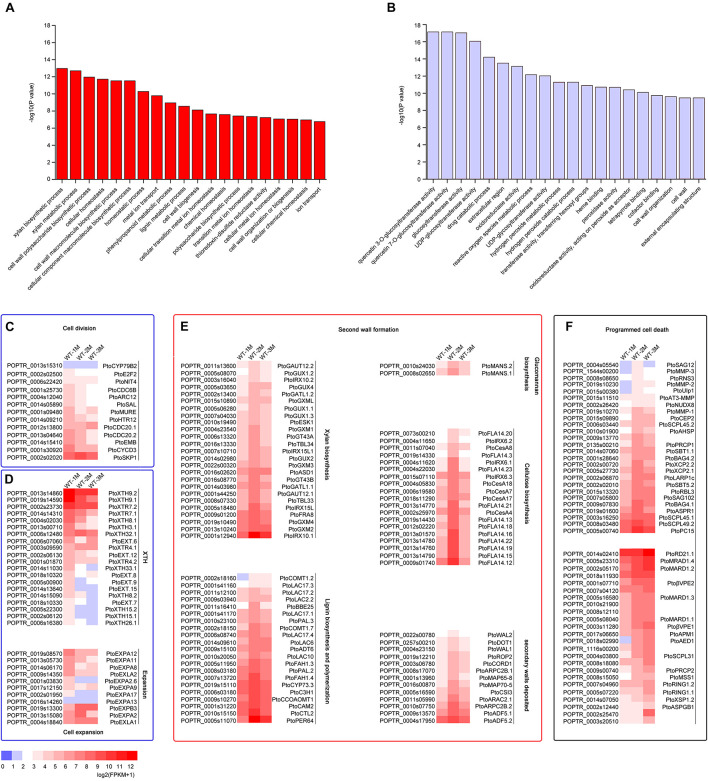
Expression patterns of DEGs involved in cell division, cell expansion, secondary wall thickening, and programmed cell death in poplar xylem. **(A)** GO enrichment analysis of DEGs in cluster 3. **(B)** GO enrichment analysis of DEGs in cluster 7. **(C)** Change in the expression of DEGs involved in cell division during xylem development. **(D)** Change in the expression of DEGs involved in cell expansion during xylem development. XTH: xyloglucan transglucosylase/hydrolase. **(E)** Expression profiles of secondary wall genes involved in cellulose, glucomannan, lignin, xylan biosynthesis, and secondary wall deposition during the secondary development of xylem in cluster 3 in *Populus*. **(F)** Expression patterns of DEGs involved in cell death during xylem development.

To identify DEGs involved in xylem development, all DEGs in the 12 clusters were subjected to GO enrichment analysis. In cluster 3, the DEGs were significantly upregulated in WT-2M plants compared with WT-1M plants, but their expression was relatively stable in WT-3M plants (Figure 2D). GO enrichment analysis revealed that most of the significant GO terms in this cluster were xylem-related GO terms, such as xylan biosynthetic and metabolic process, cell wall macromolecule and polysaccharide biosynthetic process, phenylpropanoid metabolic process, lignin metabolic process, and cell wall organization or biogenesis (Figure 3A and Supplementary Figure 1C). A total of 21 DEGs were involved in lignin biosynthesis and polymerization, 19 were involved in cellulose biosynthesis, and 25 were involved in xylan biosynthesis and secondary wall deposition, and the expression of these DEGs peaked in WT-2M plants (Figure 3E and Supplementary Table 2). This result was consistent with the rapid development of xylem in WT-2M plants, and these genes may play an important role in regulating the rapid thickening of xylem development. Downregulated DEGs in cluster 7 were mainly enriched in glucosyltransferase activity, peroxidase activity, and cell wall and organization (Figure 3B and Supplementary Figure 1), which could regulate primary cell wall formation (Supplementary Table 1).

Cluster 8 was mainly enriched in ribosome-related genes, cluster 5 was mainly enriched in vacuolar-related genes, and cluster 9 was mainly enriched in nucleus-related genes (Supplementary Figure 1). These results revealed that the ribosome, vacuolar, and nuclear-related transcripts were mainly degraded in WT-2M plants during xylem development. Plant hormone-related GO terms, especially the gibberellin biosynthetic process, were enriched in cluster 10 and cluster 11. Plant hormones play an important role in the induction of vascular element differentiation (Immanen et al., 2016). This result indicated that biosynthetic hormones and metabolic-related genes showed unique expression patterns during xylem development. In addition, cluster 12 was mainly enriched in plant biotic stress-related GO terms, cluster 1 was enriched in cell membrane-related genes 1, and cluster 2 was enriched in glyoxylate cycle and metabolic-related genes.

The mature xylem has undergone a series of cellular processes, including cell division, cell expansion, secondary wall formation, lignification, and programmed cell death. Thirteen different genes involved in the process of cell division, including *CYCD3*, *NIT4*, and *E2F2*, were expressed most strongly in WT-1M compared with WT-2M or WT-3M (Figure 3C). This result indicated that the cell division process mainly occurred in the WT-1M stage. However, cell expansion and cell wall modification-related genes were differentially expressed. A total of 31 DEGs, such as *PtoXTH8.1*/*PtoXTH8.2* (*XTH8*), *PtoEXPA8*/*PtoEXPA12* (*EXPA8*), were most strongly expressed in WT-1M plants (Figure 3D), but eight DEGs, such as *PtoEXPB3, PtoEXPA2*, *PtoXTR4.1*, and *PtoXTH32.1*, were abundantly expressed in WT-2M and WT-3M plants compared with WT-1M plants (Figure 3D and Supplementary Table 2). This result suggests that cell expansion may be important during xylem development.

We also identified 50 protease genes that may be involved in the regulation of the PCD process (Figure 3F). A total of 26 protease genes, such as *PtoXCP2.1/PtoXCP2.2* (*XCP2*), *PtoRNS3* (*RNS3*), *PtoSAG102* (*SAG102*), and *PtoSCPL45.1/PtoSCPL45.2* (*SCPL45*), were highly expressed in WT-2M plants compared with WT-1M and WT-3M plants. This result suggests that the second month may be the period during which vacuole rupture and nuclear disintegration and these genes may be involved in these processes during the early stages of PCD. A total of 24 DEGs, such as *PtoRD21.1* (*RD21B*), *Pto*β*VPE2/Pto*β*VPE1* (β*VPE*), and *PtoXSP1.2* (*XSP1*), were highly expressed in WT-3M plants compared with WT-1M and WT-2M plants and may be involved in protein degradation in the later stages of PCD.

### Analysis of Transcriptional Regulatory Network Underlying the Thickening of the Secondary Cell Wall of the Xylem

Previous studies have shown that many TFs are involved in the regulation of wood formation (Geng et al., 2020). Based on our transcriptome data, 124 TFs were upregulated in WT-2M and WT-3M plants compared with WT-1M plants, suggesting that these TFs may be potentially important regulators involved in xylem development (Supplementary Table 3). A previous study described a four-level hierarchical TRN consisting of 59 TFs in *Populus trichocarpa* (Chen et al., 2019). Among these TFs, 33 TFs, including 11 genes encoding NAC TFs and 22 genes encoding MYB TFs, were upregulated in WT-2M plants compared with WT-1M and WT-3M plants (Supplementary Table 3).

**FIGURE 4:**
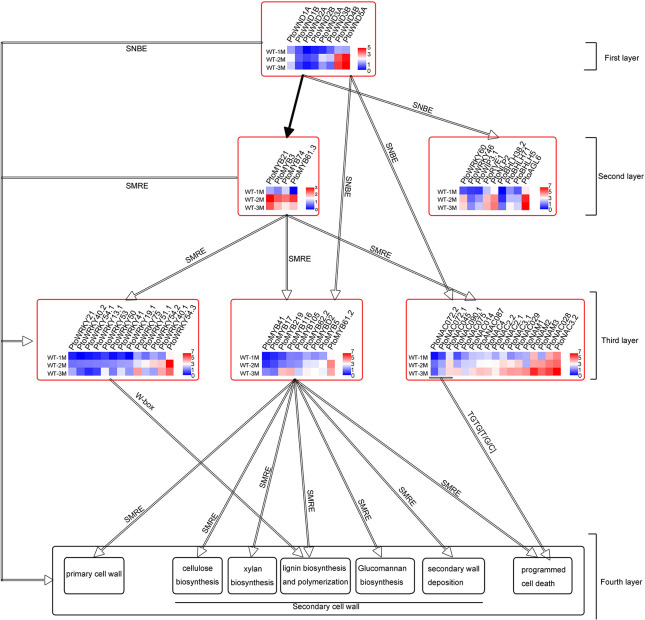
Four-layer PtoWNDs–mediated TRN depicting transcriptional regulation of cell wall biosynthesis and PCD in *P.tomentosa* during xylem development. The hollow arrows indicate predicted proteins by *cis*-acting elements; black solid arrows indicate proteins confirmed in previous research.

Two regulatory elements, namely secondary wall NAC-binding elements (SNBE) and secondary wall MYB-responsive elements (SMREs), which are considered to be key cis-acting elements, exist independently or cooperatively in the promoters of genes related to secondary wall formation (Zhong et al., 2011, 2013). The presence of SNBEs and SMREs in the 2,000-bp region of the promoter of the 116 upregulated TF genes was analyzed. The other 8 *PtoWNDs* TF genes that might act as the first switches were not analyzed. Our results showed that 9 TFs only have SNBE elements, 14 TFs only have SMRE elements, and 93 TFs have both the SNBE and SMRE (Supplementary Tables 3, 4). This suggested that xylem development-related TFs may be directly targeted by poplar (*P. tomentosa*) wood-associated NAC domain TFs (*PtoWNDs*) or MYB TFs during xylem development. Our results showed that 98 cell wall biosynthetic genes (90 secondary wall synthesis genes, 8 primary wall genes) and 47 PCD-related genes, have SNBEs or SMREs (Figure 4 and Supplementary Table 4). A conserved core *cis*-regulatory element TGTG[T/G/C] bound to *GmNAC30* (ortholog of ATAF1) and *GmNAC81* (ortholog of *AtNAC036*) was present in the promoters of PCD-related genes (Mendes et al., 2013; Reis et al., 2016). Our results showed that there was a TGTG[T/G/C] element in the promoter of 40 PCD-related genes (Supplementary Table 4), suggesting that these PCD-related genes may be regulated by *PtoNAC072.1/PtoNAC072.2* (ortholog of *GmNAC81*), or *PtoNAC045* (ortholog of *GmNAC30*).

Previous studies show that *PtrWNDs* directly binds to the SNBE sites present in the promoters of several of the WND-regulated TFs involved in secondary wall biosynthesis, cell wall modification, and PCD (Zhong et al., 2021). MYB46 and MYB83 bind to the 7 bp SMRE, ACC(A/T)A(A/C)(T/C), and directly activate a series of TFs and secondary wall biosynthetic genes. Based on previous studies and key *cis*-acting elements SNBE, SMRE, W-box or TGTG[T/G/C], the 60 TFs, 98 cell wall CW biosynthetic genes, and 47 PCD genes (Supplementary Table 4) were used to construct a four-layered *PtoWNDs*–mediated TRN for demonstrating genes related to cell wall biosynthesis and PCD in *P. tomentosa*. Eight *PtoWNDs*, which may act as master switches involved in xylem development, were in the top (first layer) of this TRN. Based on the key cis-acting elements (SNBEs), 13 TFs including 4 MYB TFs, which may act as the second master switches involved in xylem development, were in the second layer (Figure 4). Based on the key cis-acting elements (SMREs), 9 MYB TFs, which may act as the third master switches involved in xylem development, were in the third layer (Figure 4). In addition, 17 NAC and 13 WRKY TFs, both SMREs and SNBEs, were in the third layer (Figure 4). Based on the key cis-acting elements TGTG[T/G/C], the PCD-related genes might be targeted by *PtoNAC072.1/PtoNAC072.2* (ortholog of *GmNAC81*), and *PtoNAC045* (ortholog of *GmNAC30*) (Figure 4 and Supplementary Table 4). PCD-related genes also have SNBE or SMRE, suggesting that these genes may be regulated at multiple levels. A total of 14 cell wall biosynthetic genes without SNBE elements, 84 cell wall biosynthetic genes with SNBEs and SMREs, and 40 PCD-related genes with TGTG[T/G/C] elements may be targeted by *PtoWNDs* and MYBs and were in the fourth layer (Supplementary Table 4).

**FIGURE 5:**
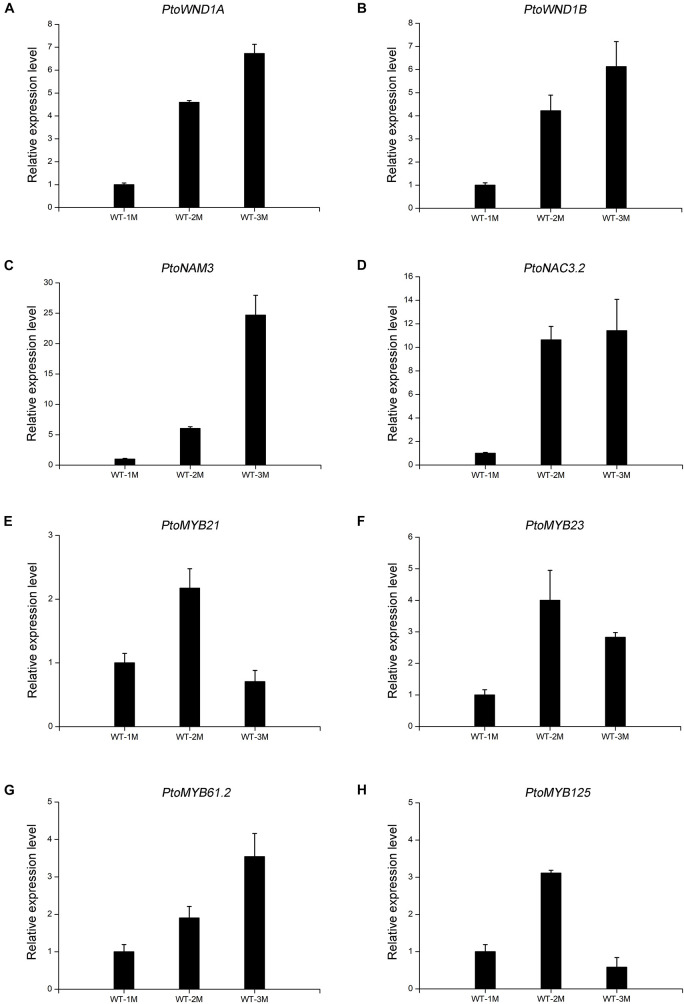
The expression levels of TFs involved in xylem development by qRT-PCR in WT-1M, WT-2M, and WT-3M plants **(A–H)**. The expression levels of *PtoWND1A, PtoWND1B, PtoNAM3, PtoNAC3.2, PtoMYB21, PtoMYB23, PtoMYB61.2*, and *PtoMYB125*, respectively. The expression was normalized to the *actin* gene. Relative expression levels of candidate genes were calculated by the 2^–ΔΔ*Ct*^ method. All values shown are means ± *SD* (*n* = 3).

To verify the reliability of the transcriptome data, qRT-PCR analysis was performed. Transcript levels of the eight genes selected, including four NAC TFs (*PtoWND1A*, *PtoWND1B*, *PtoNAM3*, and *PtoNAC3.2*) and four MYB TFs (*PtoMYB21*, *PtoMYB23*, *PtoMYB61.2*, and *PtoMYB125*), were measured in WT-1M, WT-2M, and WT-3M plants using qRT-PCR. Expression patterns were similarly based on RNA-seq and qRT-PCR, thus validating the transcriptome data (Figure 5).

### Proteomic Analysis of Protein Expression During Xylem Development

**FIGURE 6:**
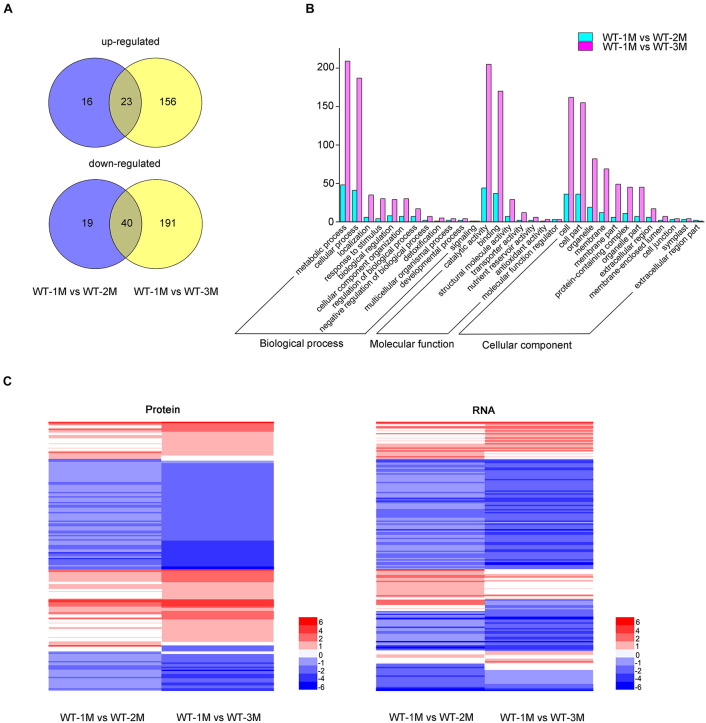
Proteomic analysis of differentially expressed proteins involved in xylem development in *P. tomentosa*. **(A)** Numbers of upregulated and downregulated proteins in WT-1M vs. WT-2M and WT-1M vs. WT-3M. *P* < 0.05, fold change ≥ 2 or ≤ –0.5. **(B)** Numbers of differentially expressed proteins of “Biological process,” “Cellular component,” and “Molecular function” in different functional categories in WT-1M vs. WT-2M and WT-1M vs. WT-3M. **(C)** Hierarchical cluster analysis of differentially expressed proteins of WT at three different months. The color scale bar on the right indicates the expression proteins and mRNA level. The values given in the legend are log2 (fold change).

To determine whether the protein levels of genes related to xylem development were altered, we conducted a proteomic analysis of the stem xylem at different stages. A total of 3,657 proteins were detected in the proteome data sets from WT-1M, WT-2M, or WT-3M plants. We identified 445 proteins with significantly different expression levels (fold change ≥ 2 or ≤ –0.5, and corrected *P*-value < 0.05) in at least one of the three time points (Figure 6A). These differential proteins could all be detected in the transcriptome. Metabolic process and cellular process in “Biological process”; cell, cell part, membrane and organelle related genes in “Cellular component”; and binding and catalytic activity in “Molecular function” were enriched by GO classification (Figure 6B). This result is consistent with the GO analysis of the transcriptome (Figures 2B, 6B).

Compared with the transcriptome data, the expression patterns of 121 differentially expressed proteins were consistent with mRNA abundances (Figure 6C). Combining the transcriptome and proteome datasets, we found that the expressions of 72 proteins have different expression patterns at all three time points (Figure 6C and Supplementary Table 5). We found that these proteins have post-transcriptional modification (PTM) sites by analyzing the amino acid sequences of these proteins through the Plant PTM Viewer (Supplementary Table 5). These results suggested that PTMs might be widely involved in lignification development.

## Discussion

### Transcriptomic Networks Underlie the Regulation of Cell Division, Expansion, Secondary Cell Wall Thickening, and Programmed Cell Death in Xylem Development

Plant vascular tissue is mainly composed of xylem, phloem, protocambium vascular cambium. Our results showed that xylem wall thickening was rapid in *P. tomentosa*, and the rapid growth of xylem cells and xylem cell PCD and the rapid thickening of secondary walls was a synergistic process. Although initially rapid, the increase in both the number of xylem cells and the thickness of the secondary wall later plateaued. The maturation of the plant xylem involves cell division, expansion, cell wall thickening, and PCD. There were 12 cell cycle-related genes, including CYCD3;1, CDC20-like protein, E2F2, CDC20, CDC6B, and CYCD3, which were involved in the induction of cell division (Bomer et al., 2021), were highly expressed in WT-1M compared with WT-2M and WT-3M plants (Figure 3C), suggesting that they were involved in cell division during xylem development. Recent studies have confirmed that XTHs contribute to the formation of secondary cell walls of vascular tissues and are thought to be important regulators of primary wall expansion (Li et al., 2013; Kushwah et al., 2020). In our study, XTHs have enriched in clusters 7 and their expression was lower in WT-2M and WT-3M plants compared with WT-1M plants (Figure 3D). Expansions were involved in cell expansion in all plant tissues and have been isolated from the secondary xylem (Gray-Mitsumune et al., 2004). In our study, *PtoXTR4.1*, *PtoEXPB3*, *PtoPL1/3/5*, and *PtoPAE9*, which encode pectin/pectate lyase, or expansion-related proteins were differentially expressed in WT-1M, WT-2M, and WT-3M plants (Figure 3D and Supplementary Table 2). Poplar xylan synthesis genes were highly expressed in developing wood and were specifically upregulated in the secondary wall forming zone (Sundell et al., 2017). In our study, the xylan synthesis genes IRX9, FRA8, IRX8, and GXM1/2/3/4 were highly expressed in WT-2M plants relative to WT-1M or WT-3M plants (Figure 4B). The expression patterns of genes related to cellulose biosynthesis, lignin biosynthesis, and secondary wall deposition were consistent with those of genes related to xylan biosynthesis. These expression patterns were also consistent with the rapid thickening of the secondary wall in WT-2M plants relative to WT-1M plants.

After the end of lignification, TEs, and fiber cells undergo PCD. The PCD process of TEs includes the degradation of organelles and is accompanied by the degradation of protoplasts and the degradation of partially unlignified secondary walls (Escamez and Tuominen, 2014). In our study, the candidate proteases showed different expression patterns. For example, vacuolar protease XCP2 (*PtoXCP2.1/PtoXCP2.1*) and nuclease RNS3 (*PtoRNS3*) were highly expressed in WT-2M compared with WT-1M and WT-3M and might be involved in DNA degradation and vacuole rupture (Figure 3F). However, *PtoRD21.1*, *Pto*β*VPE2/Pto*β*VPE1*, and *PtoAED1* were highly expressed in WT-3M compared with WT-1M and WT-2M, which might be involved in the degradation of cell components in the later stages of PCD in xylem development. The results of the GO enrichment analysis results revealed that the expression of genes related to the ribosome, vacuole, nucleus, and other organelles were downregulated in WT-2M plants compared with WT-1M or WT-3M plants (Supplementary Figure 1). These results indicated that the different expression patterns of the PCD protease genes play a key role in the degradation of organelles during the rapid thickening of the xylem and the degradation of cell components in the later stage. These results also showed that genes varied in their temporal expression patterns.

SWINGER proteins (SWNs) have been shown to activate various downstream TFs, including SND2, SND3, MYB20, MYB42, MYB43, MYB46, MYB52, MYB54, MYB58, MYB63, MYB69, MYB83, MYB85, MYB103, and KNAT7 (Zhong et al., 2008), involved in the transcriptional regulation of secondary wall biosynthesis. Our transcriptome data revealed that 35 TFs homologous to *Arabidopsis*, including SND1, SND3, XND1, MYB46, MYB83, MYB103, MYB20, MYB42, MYB50, MYB52, MYB54, MYB59, MYB85, MYB69, and MYB61, were highly expressed in WT-2M plants compared with WT-1M and WT-3M plants (Supplementary Table 3). This suggested that these TFs regulate the rapid thickening during early xylem development by participating in the rapid synthesis of the secondary wall.

In this study, 50 TFs related to xylem development with unknown functions were identified and integrated into this TRN based on the key *cis*-acting elements SNBEs, SMREs, W-box, and TGTG[T/G/C]. We also elucidated the regulatory network of TFs with known functions in this TRN. *Arabidopsis AtMYB61*, *Poplar* MYB61 (POPTR_0015s09430) positively regulated the lignin and cellulose biosynthesis (Romano et al., 2012; Li et al., 2018). Promoter analysis showed that *PtoMYB61.2* (POPTR_0005s00340) have SNBEs and SMREs, *PtoMYB61.3* (POPTR_0002s18700) only has SNBE elements and is an ortholog of *AtMYB61*, suggesting that *PtoMYB61.3* may be targeted by *PtoWNDs* and act as the second master switches involved in the lignin and cellulose biosynthesis (Supplementary Figure 2). In previous studies, *Arabidopsis* AtWRKY13 regulated the formation of secondary cell walls, but its upstream regulatory factors remained unclear (Li et al., 2015). Promoter analysis showed that *PtoWRKY13.1*, homologous to Arabidopsis *AtWRKY13*, has SNBEs and SMREs, suggesting that *PtoWRKY13.1* may be targeted by *PtoWNDs* and *MYBs* (Supplementary Figure 2).

### Integrated Transcriptome and Proteome Analysis Reveals Post-transcriptional Modifications During Xylem Development

Post-transcriptional modification of proteins plays a key role in many biological processes in plants. Over the past decade of research, the PTMs of several proteins have been associated with lignification (Morse et al., 2009; Zhang et al., 2014; Nasir et al., 2018). We found that 72 proteins might be regulated by PTMs during xylem development using Plant PTM Viewer prediction (Supplementary Table 5). Phospho-proteomic analysis by mass spectrometry in stem differentiating xylem (SDX) revealed that the phosphorylation of PtrAldOMT2 (homologous to AtOMT1) was mediated by kinases in the SDX protein extract (Wang et al., 2015). In addition, the phosphorylation of PtrAldOMT2 led to the phosphorylation of MAT2 in the stem-differentiating xylem. Previous studies have also shown that CPK28 targets MATs (MAT1, MAT2, and MAT3) for degradation by the 26S proteasome pathway, and thus affects ethylene biosynthesis and lignin deposition in *Arabidopsis* (Chen et al., 2013; Jin et al., 2017). In our study, PtoMAT2 (homologous to AtMAT2) was differentially expressed in the transcriptome and proteome, indicating that it may be phosphorylated and degraded by the 26S proteasome pathway. In addition, differences in the expression of several PCD proteases, such as PtoXCP2.1, PtoSBT1.1, PtoSCPL25, and PtoSCPL46, were observed in transcriptome and proteome, suggesting that they may be regulated by PTMs. The presence of these PTMs in SDX suggests that PTMs were regulated following the differentiation process from cell division to cell death during xylem development.

## Materials and Methods

### Plant Materials and Growth Conditions

The poplars used in the study were *P. tomentosa* 741. The plants were grown on solid Murashige and Skoog (MS) medium (Caisson, United States) in a growth chamber with a 14 h light/10 h dark photoperiod at 25°C. We selected the stems from 1, 2, and 3-month-old poplar plants showing good growth for analyses.

### Stem Histochemistry

We selected approximately 5-mm stem sections from the roots up to the fourth stem node for paraffin sectioning. To observe the development and changes in the xylem of *P. tomentosa* at three different ages (1, 2, and 3 months). The specific experimental methods followed those of a previous study (Han et al., 2019). Micrographs of sections from 9 to 12 specimens were examined. Statistical differences were determined using Student’s *t*-test.

### Transmission Electron Microscopy

Roots up to the fourth stem node of *P. tomentosa* were collected for TEM analysis at three different ages (1, 2, and 3 months). The sample preparation method of TEM follows that of Han et al. (2019). Sections were examined under an H-7650 transmission electron microscope at 80 kV (Hitachi, Japan) and images were taken using an 832 charge-coupled device camera (Gatan). Cell wall thickness was determined as the mean value of four measurements taken perpendicularly across the wall of each cell using ImageJ software^1^. Statistical differences were determined using Student’s *t*-test.

### Scanning Electron Microscopy

A new sharp double-sided blade was used to cross-cut the material with the roots of *P. tomentosa* tissue-cultured seedlings in different months up to the fourth stem node 1∼2 mm thick. The sample preparation method of SEM followed that of a previous study (Zhang et al., 2014). Samples coated with gold particles were observed using a scanning electron microscope (Quanta200), and the accelerating voltage was 15 kV.

### Transcriptome Analyses

The cortex and phloem were peeled off the stems of *P. tomentosa* at 1, 2, and 3 months of age, and the xylem was collected for transcriptional analyses. There were three biological replicates for each sample. We performed transcriptome sequencing according to the previous study (Li et al., 2021). Raw RNA-seq reads were available at the NCBI Sequence Read Archive (SRA) under the accession number PRJNA722127.

Furthermore, *P*-values were adjusted and the false discovery rate was calculated using the Benjamini and Hochberg approach. A corrected *P*-value of < 0.05 and a log2 fold change ≥1 or ≤–1 were the thresholds for significant differential expression. GO enrichment analyses of the DEGs were performed using the GOseq R package (Wang et al., 2010). GO terms with a *P*-value < 0.005 were considered significantly enriched. Genes were clustered into 4 groups, then into 12 clusters, according to changes in gene expression among the 3 months.

### Quantitative Real-Time PCR

Total RNA was extracted from the xylem of poplar stems in different months using the RNAprep Pure Plant Kit (Tiangen Biotech, Beijing, China) following the instructions of the manufacturer. Quantitative real-time PCR (qRT–PCR) was performed using gene-specific primers and FastFire qPCR PreMix (Probe) (SYBR Green; Tiangen Biotech, China) on a CFX Maestro software Real-Time System (Bio-Rad, Hercules, CA, United States). Eight genes including NAC and MYB TFs (*PtoWND1A*, *PtoWND1B*, *PtoNAM3*, *PtoNAC3.2*, *PtoMYB21*, *PtoMYB23*, *PtoMYB61.2*, and *PtoMYB125*) were validated, and the primers are shown in Supplementary Table 6. The PCR conditions and processes followed those of Li et al. (2021). The *P. tomentosa actin* gene was used as the internal control. All reactions were run in triplicate for each sample. Relative expression levels of candidate genes were calculated by the 2^–ΔΔCt^ method.

### Proteome Analysis

The cortex and phloem were peeled off the stems of 1, 2, and 3-month-old *P. tomentosa* from the roots up to the fourth stem node. Proteomic methods followed those of a previous study (Zhu et al., 2014). We deposited our proteome data set in ProteomeXchange under the accession number PXD025418. Significant enrichment of GO terms and Kyoto Encyclopedia of Genes and Genomes pathways for differentially expressed proteins (fold change ≥ 2 or fold change ≤ 0.5) was determined using Fisher’s exact test (*P* ≤ 0.05). There were three biological replicates for each sample. The PTM site of proteins was predicted using Plant PTM Viewer (VIB Bioinformatics Core, Belgium)^2^.

## Data Availability Statement

The datasets presented in this study can be found in online repositories. The names of the repository/repositories and accession number(s) can be found in the article/Supplementary Material.

## Author Contributions

CZ and HLu designed the experiments, analyzed the data, and wrote the manuscript. CZ, JZ, YL, XL, XG, HLi, DL, and HLu performed the experiments. CZ performed the bioinformatics analysis. All authors read and approved the manuscript.

## Conflict of Interest

The authors declare that the research was conducted in the absence of any commercial or financial relationships that could be construed as a potential conflict of interest.

## Publisher’s Note

All claims expressed in this article are solely those of the authors and do not necessarily represent those of their affiliated organizations, or those of the publisher, the editors and the reviewers. Any product that may be evaluated in this article, or claim that may be made by its manufacturer, is not guaranteed or endorsed by the publisher.

## Footnotes

1
https://imagej.nih.gov/ij

2
https://www.psb.ugent.be/webtools/ptm-viewer
