# Supplementary figures and images for: Integrated Transcriptomic and Proteomic Analysis in the Roadmap of the Xylem Development Stage in Populus tomentosa
Source: Front Plant Sci. 2021 Nov 4;12:724559. doi: 10.3389/fpls.2021.724559 (PMC8600231; doi:10.3389/fpls.2021.724559)

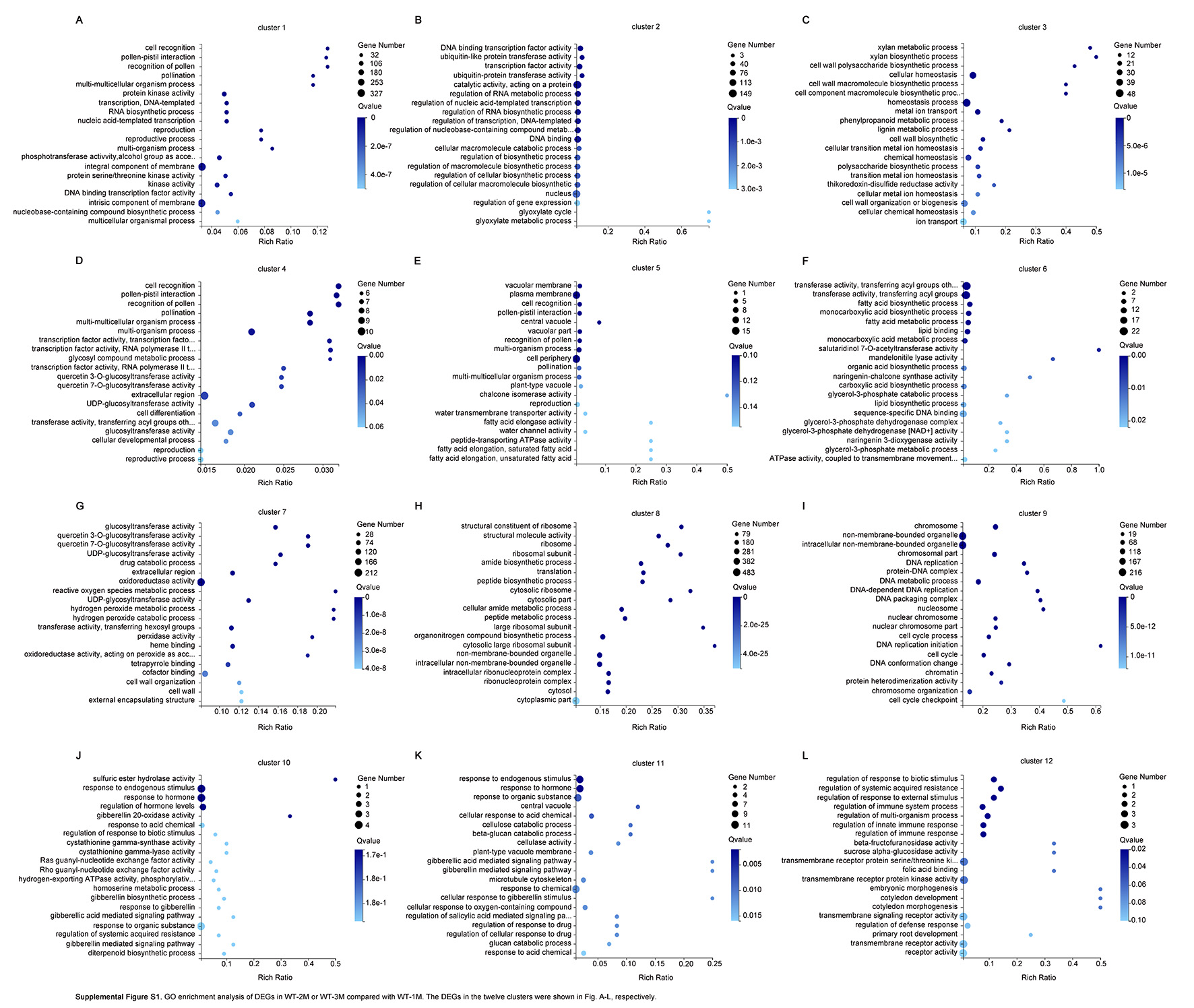

Supplement: Supplementary file 1 [file Image_1.JPEG]

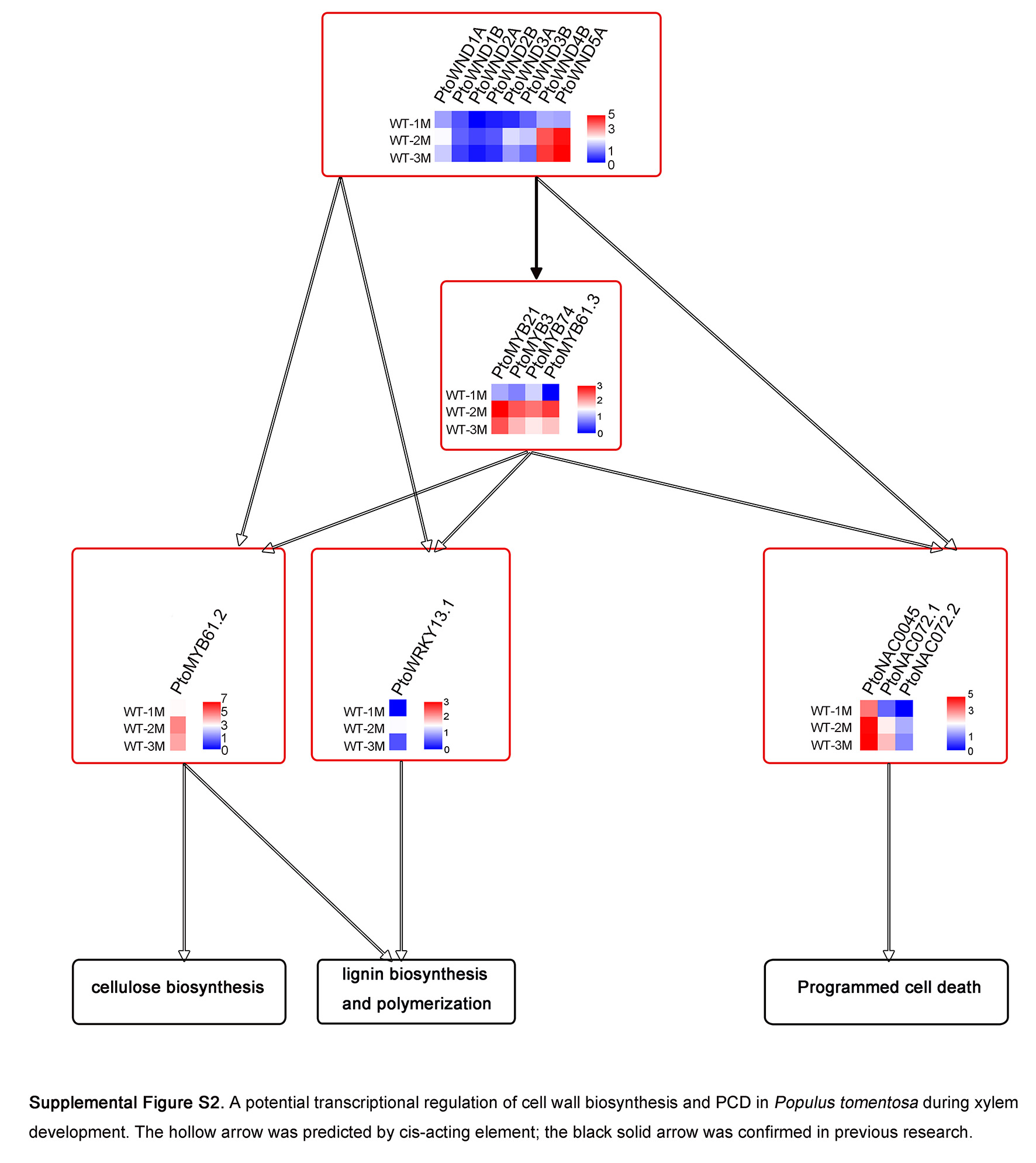

Supplement: Supplementary file 2 [file Image_2.JPEG]
